# Comparative assessment of enamel remineralisation on the surface microhardness of demineralized enamel - an *in vitro* study

**DOI:** 10.7717/peerj.14098

**Published:** 2022-10-07

**Authors:** Bhavika Bhavsar, Mary Vijo, Pranjely Sharma, Tulika Patnaik, Mohammad Khursheed Alam, Santosh Patil

**Affiliations:** 1 Department of Conservative Dentistry and Endodontics, Rkdf Dental College and Research Centre, Bhopal, Madhya Pradesh, India; 2 Orthodontics, Preventive Dentistry Department, College of Dentistry, Jouf University, Sakaka, Saudi Arabia; 3 Center for Transdisciplinary Research (CFTR), Saveetha Dental College, Saveetha Institute of Medical and Technical Sciences, Saveetha University, Chennai, India; 4 Department of Public Health, Faculty of Allied Health Sciences, Daffodil lnternational University, Dhaka, Bangladesh; 5 Department of Oral Medicine and Radiology, New Horizon Dental College and Research Institute, Sakri, Bilaspur, India

**Keywords:** Carious lesions, Microhardness, Fluoride, Remineralisation, Bioglass, Hydroxyapatite, Calcium phosphate, Demineralisation

## Abstract

**Objective:**

The main objective of the study was to compare two different remineralising materials containing casein phosphopeptide-amorphous calcium phosphate, bioactive glass on enamel surface microhardness.

**Materials and Methods:**

Thirty premolars were used for specimen preparation. Group 1 (the control group) consisted of intact enamel samples, group 2: CPP-ACPF (Tooth Mousse Plus), group 3: bioenamel remineralising gel (Prevest DenPro). All specimens were subjected to demineralisation except the control group, followed by which remineralising agents were applied. A universal hardness tester was used to assess the surface microhardness of all samples. Results were analysed using one-way ANOVA test and comparison was analysed using Scheffe’s *post hoc* least significant difference (LSD) test.

**Results:**

Both remineralising agents used in groups 2 and 3 have shown significant outcome in terms of improving the surface microhardness in comparison with the control group. Group 2 increased the enamel hardness by 8.34 where *P* = 0.023 whereas group 3 increased the hardness by 5.87, where *P* = 0.01.

**Conclusion:**

Group 2 has a superior hardness value than group 3; however, no statistically significant results were obtained between both the groups.

## Introduction

Enamel is the outermost layer of the tooth which undergoes demineralization by increased intake of acidic and sugar-containing foods where salivary ions and antibacterial agents promote remineralization. To maintain continuous remineralization and demineralization balance is difficult. If the balance is disturbed for a longer period of time, it ultimately leads to initial carious lesions (Salinovic et al., 2021).

Dental caries is one of the common etiologies for tooth structure loss which is easy to detect and cure at the initial stage. So, it is important to prevent dental caries at the initial stage itself rather than going for complex treatment modalities.

So far the treatment modality of carious tooth is excavation followed by restoration. The minimally invasive approach has evolved which promotes detecting and treating the initial lesions earlier, and is mainly concerned on prevention (Rahiotis & Vougiouklakis, 2007).

The minimally invasive dentistry involves procedures which avoid maximum destruction of dental tissues. One such modality is the remineralization of initial, non-cavitated lesions, avoiding conventional treatment (Salinovic et al., 2021; Villalobos-Rodelo et al., 2013). Remineralizing agents with casein phosphopeptide-amorphous calcium phosphate and bioactive glass has been used for remineralization of enamel with sufficient outcomes (Palaniswamy et al., 2016).

Casein phosphopeptide-amorphous calcium phosphate (CPP-ACP) is a constituent of nanocomplexes of milk protein which maintains a supersaturated state with minerals to promote remineralisation, and also hinders cariogenic bacterial colonization. It also has anticariogenic potential where these nanocomplexes from CPP-ACP gets incorporated into the dental plaque (Reynolds, 1998).

GC Tooth Mousse is one such agent which is available as CPP–ACPF paste (GC Tooth Mousse Plus; GC Corporation, Tokyo, Japan) contains 0.09% of fluoride. CPP–ACPF have a greater remineralisation potential than CPP–ACP (Reynolds et al., 2008; Soares et al., 2017).

Bio Enamel remineralising gel (Prevest Denpro Ltd, Jammu, India) is another remineralizing agent which enhances the remineralization of enamel. It could be indicated in areas of de-mineralized tooth structure, in case of post-bleaching sensitivity and sensitivity caused due to non-carious lesions and after oral prophylaxis. Very scarce information is available regarding the remineralisation of carious lesions using bioenamel in literature.

Until now, studies comparing the remineralisation potential of CPP-ACPF and the bioenamel remineralising gel are scarce. Hence, this *in vitro* study was done to evaluate the remineralising potential of the above mentioned agents on demineralised enamel surface by surface microhardness (SMH) analysis.

## Materials and Methods

Ethical approval was obtained from the ethical committee before commencing the study (RKDF/DC/PG/2022/17688). A total number of 30 human premolars extracted for orthodontic purposes were selected for the study. Teeth with caries, previous restorations, any altered surface morphology, stains, craze lines, white spot lesions, developmental anomalies, hypoplastic, hypocalcified teeth and erosive lesions were excluded from the study. The teeth were cleaned properly and were stored in artificial saliva (0.04wt% NaCl, 0.04wt% KCl, 0.09wt% CaCl2·2H2 O, 0.069wt% NaH2 PO4·2H2, 0.008wt% MgCl2·6H2O, 0.1wt% glucose, 0.05wt% urea,10 ml water, 7wt% PEG 6000 0.15 mg methyl-p-hydroxybenzoate, 0.01wt% ascorbic acid, with a pH set at 6.8) at room temperature.

Teeth were sectioned 1 mm just below the cementoenamel junction (CEJ) using a slow speed diamond disc. Only the crowns were used in this study. The samples were stored in thymol solution (0.1%) till the procedure was carried out. Moulds were prepared into which acrylic resin was poured. The sectioned crown was placed parallel to the horizontal plane in the resin mould with the buccal surface upward and exposed. The exposed buccal surface was made flat and polished using Mintech 233 (Presi, Le Loche, Switzerland, Europe) polishing machine (300 rpm), with water coolant to perform microhardness test using the formula:

${\rm Sample}\,{\rm size \ \!(n) = }\displaystyle{{(r + 1)} \over r}\displaystyle{{{{(SD)}^2}{{({z_{\alpha /2}} + {z_\beta })}^2}} \over {{d^2}}}$

sample size was calculated as 10 for each group. For 3 groups total sample size should be 3 × 10 = 30 samples.

30 specimens were randomly divided into 3 groups of 10 samples each (*n* = 10).

Group A: Sound tooth without any treatment (control);

Group B: CPP-ACPF paste Tooth Mousse Plus;

Group C: Bioenamel remineralising gel.

**Figure 1:**
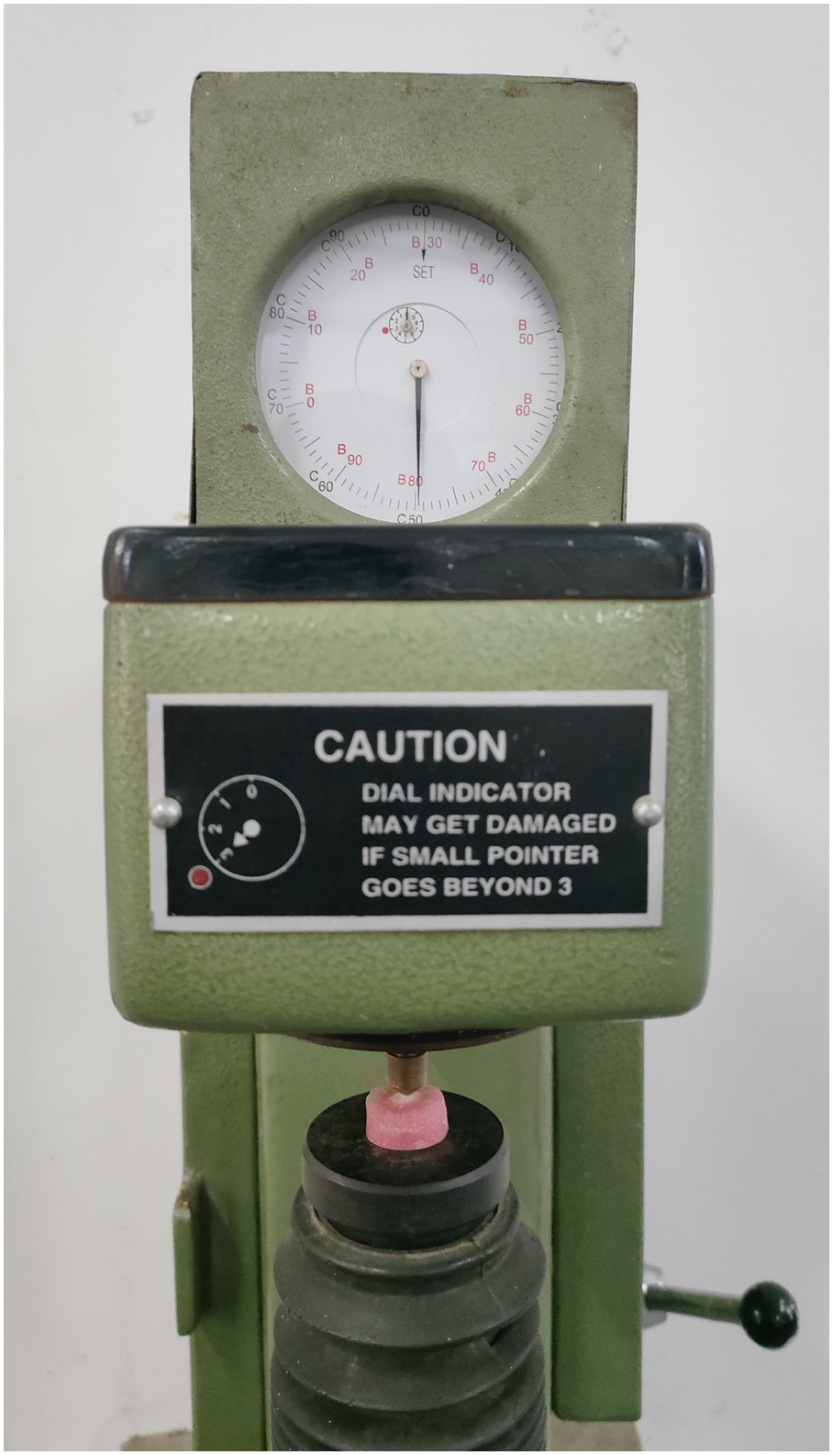
Brinell hardness testing machine with sample loaded.

**Figure 2:**
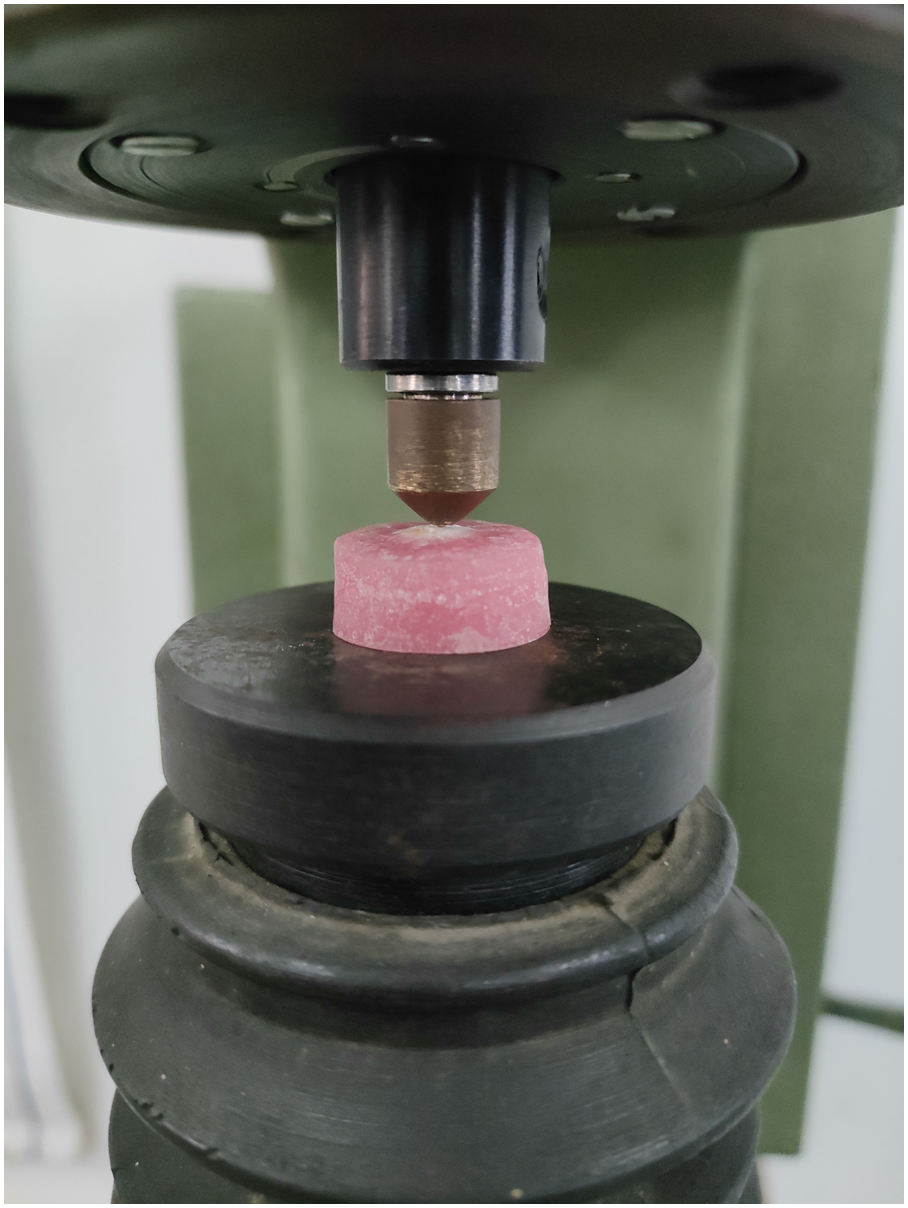
Microhardness indentation on the sample.

Microhardness of each sample was measured using universal hardness testing machine at three different stages: baseline, after demineralization, and after remineralization as shown in the Fig. 1. A total of 50N of standard load was applied for 15 s to measure the microhardness, as proposed by Farooq et al. (2019). Three indents were made on each sample as shown in the Fig. 2 and the mean value was calculated.

**Figure 3:**
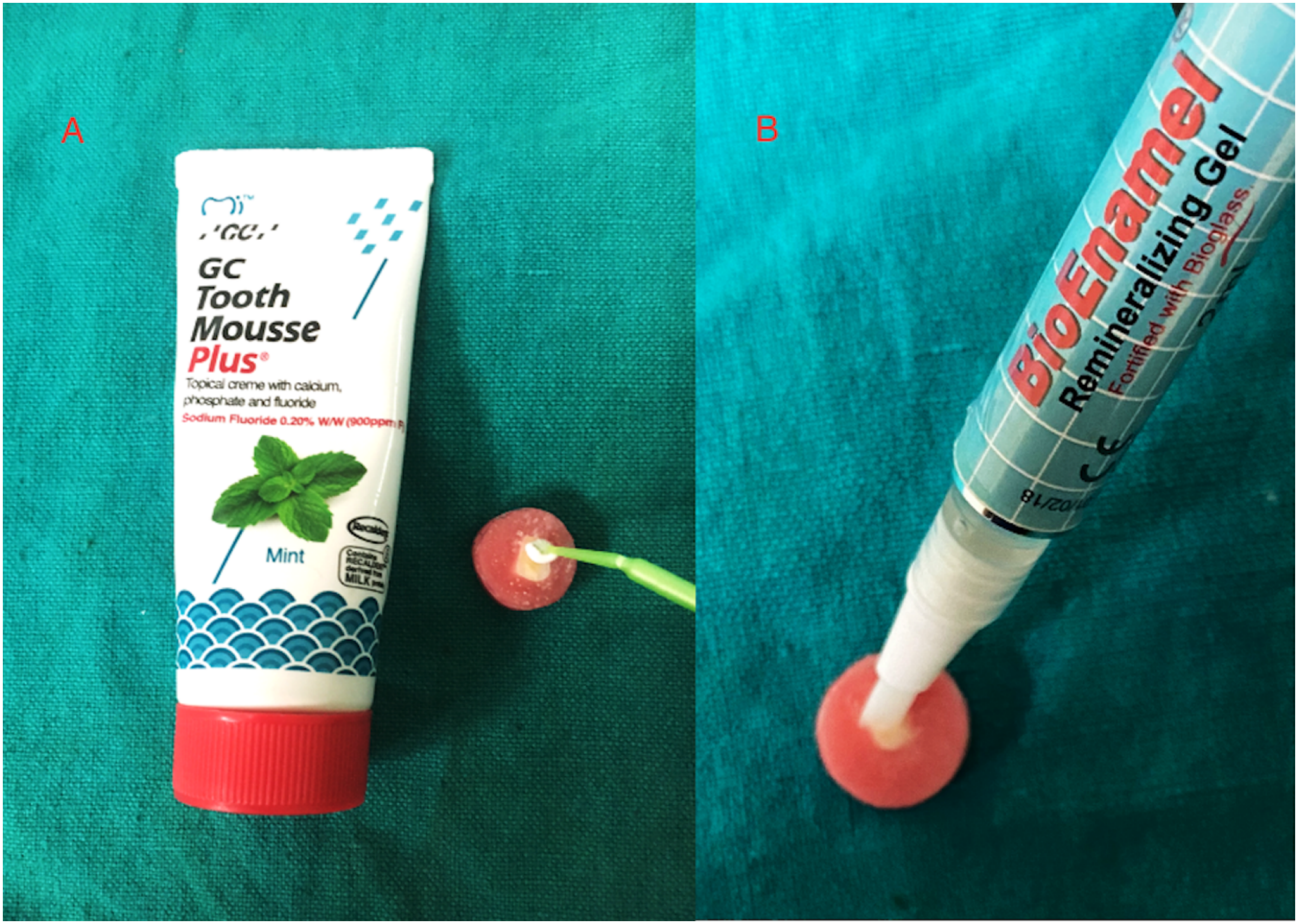
Remineralising agent application on the sample. (A) CPP ACPF Tooth Mousse Plus; (B) Bioenamel remineralising agent.

A total of 37% phosphoric acid (Prevest Denpro Ltd, Jammu, India) was applied on the buccal surface of the sample for 3 min to bring about the demineralisation (Salinovic et al., 2021). The samples were washed, air-dried and the microhardness was measured again with the indenter as previously. The samples were stored in artificial saliva to prevent dehydration for the next 24 h. Remineralizing agents were then applied using a applicator brush (3M ESPE, St. Paul, IL, USA) as shown in Fig. 3.

Group 1: Control group – immersed in artificial saliva (10 ml) no treatment done, and the solution was refreshed twice daily.

Group 2: treated with GC Tooth Mousse Plus for 5 min every 12 h for 14 days, paste was wiped without rinsing and stored in artificial saliva.

Group 3: treated with Bioenamel remineralising gel for 5 min every 12 h for 14 days and immersed in artificial saliva.

Surface microhardness (SMH) was measured again to evaluate the remineralization capability of each remineralising agent, and the changes were statistically analysed.

### Statistical analysis

Data were analysed using mean and standard deviation. Intergroup comparison for microhardness changes due to demineralization and remineralization was done using one-way ANOVA test. Scheffe’s *post hoc* least significant difference (LSD) test was used to perform individual pairwise comparison. Independent sample test were used to bring about the comparison with control group. Statistical significance was denoted when *P* < 0.05.

## Results

**Figure 4:**
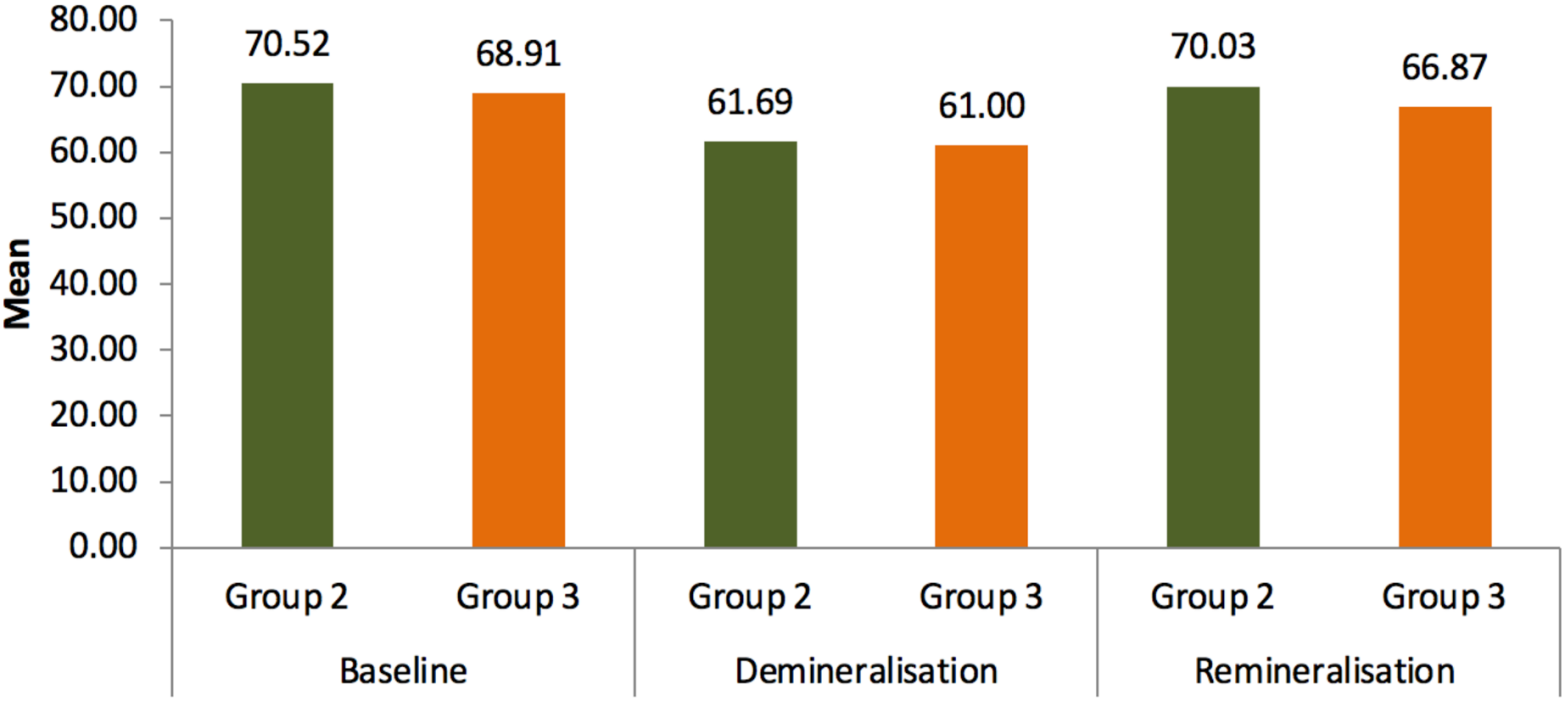
Mean comparison of microhardness between group 2 and group 3.

Results of microhardness test were given in the table. Statistical analysis showed that the readings of group 2 (Tooth Mouse Plus) and group 3 (bioenamel remineralising gel) increased after 14 days of treatment where group 2 has shown *P* = 0.023 S and group 3 *P* = 0.01 S when compared to the readings after demineralization where group 2 had *P* = 0.015 S and group 3 had *P* = 0.021 S when compared to the baseline values. Both remineralising agents used in groups 2 and 3 have shown significant outcome in means of improving the surface microhardness in comparison with the control group as given in Tables 1 and 2. Group 2 has improved the surface hardness by 8.34 where *P* = 0.023 whereas group 3 has increased the hardness by 5.87 where *P* = 0.01 as shown in Tables 3 and 4. Although extreme change in surface hardness was observed in group 2 followed by group 3, no statistically significant results were obtained between both the groups as mentioned in Table 5 and Fig. 4.

**Table 1:** Mean comparison of microhardness between control group & group 2 (Remineralisation) and the control group & group 3 (Remineralisation).

Group	MEAN	SD	Mean difference	t value	*P* value
Group 1-Control	72.91	6.73	2.88	0.917	0.371 NS
Group 2-Remineralisation	70.03	7.31			

Group 1-Control	72.91	6.73	6.04	2.672	0.001 S
Group 3-Remineralisation	66.87	9.23			

**Note:**

Statistical Analysis: Independent sample t test. S: Significant at the 0.05 level. NS, Not significant.

**Table 2:** Mean comparison of microhardness of the three different stages of group 2 and group 3.

		MEAN	SD	F value	*P* value
Group 2	Baseline	70.52	7.30	4.087	0.028 S
	Demineralisation	61.69	8.61		
	Remineralisation	70.03	7.31		
Group 3	Baseline	68.91	9.03	5.781	0.001 S
	Demineralisation	61.00	10.82		
	Remineralisation	66.87	9.23		

**Note:**

Statistical Analysis: ANOVA one way test. S: Significant at the 0.05 level. NS, Not significant.

**Table 3:** Mean comparison of microhardness of the three different stages of group 2.

Group 2	MEAN	SD	Mean difference	*P* value
Baseline	70.52	7.30	8.83	0.015 S
Demineralisation	61.69	8.61		

Baseline	70.52	7.30	0.49	0.990 NS
Remineralisation	70.03	7.31		

Demineralisation	61.69	8.61	8.34	0.023 S
Remineralisation	70.03	7.31		

**Note:**

Statistical Analysis: Scheffe’s *post hoc* test. S: Significant at the 0.05 level. NS, Not significant.

**Table 4:** Mean comparison of microhardness of the three different stages of group 3.

Group 3	MEAN	SD	Mean difference	*P* value
Baseline	68.91	9.03	7.91	0.021 S
Demineralisation	61.00	10.82		

Baseline	68.91	9.03	2.04	0.896 NS
Remineralisation	66.87	9.23		

Demineralisation	61.00	10.82	5.87	0.01 S
Remineralisation	66.87	9.23		

**Note:**

Statistical Analysis: Scheffe’s *post hoc* test. S: Significant at the 0.05 level. NS, Not significant

**Table 5:** Mean comparison of microhardness between group 2 and group 3.

	Group	MEAN	SD	Mean difference	t value	*P* value
Baseline	Group 2	70.52	7.30	1.61	0.438	0.666 NS
	Group 3	68.91	9.03			
Demineralisation	Group 2	61.69	8.61	0.69	0.158	0.876 NS
	Group 3	61.00	10.82			
Remineralisation	Group 2	70.03	7.31	3.16	1.848	0.144 NS
	Group 3	66.87	9.23			

**Note:**

Statistical Analysis: Independent sample t test. S: Significant at the 0.05 level. NS, Not significant.

## Discussion

In this study, microhardness of enamel surface of a tooth has been evaluated to determine the extent of remineralisation. The procedure adopted for preparation has been used by many authors.

Non-invasive intervention plays an important role to transform an active lesion to an inactive state. Minimally invasive techniques has to be followed to restore lost tooth structure without compromising the tooth.

Acidic components released by bacteria from the dental plaque leads to destruction of inorganic components of enamel by a chemical process called demineralization (Neel et al., 2016). Remineralisation involves diffusion of salivary ions like calcium, phosphate and fluoride that builds a fluorapatite layer on crystal which is acid resistant, hypermineralised that act as a remineralised nuclei (Featherstone, Duncan & Cutress, 1979).

As suggested by Sorozini, Perez & Rocha (2018) the artificial demineralised lesion has been created using 37% phosphoric acid which led to a significant mineral loss, lowering the hardness values of each group seems to be sufficient, as complete simulation of oral conditions outside the oral cavity is almost impossible because of the salivary flow and some behavioural changes (Molaasadolah et al., 2017).

In this study, two commercially available remineralising agents were evaluated using microhardness testing. Enamel surface layer plays a significant role in caries progression, hence evaluation of such surface change is relevant. SMH measurement is used to determine the hardness of enamel. SMH indentation is relevant for materials with fine microstructure, non-homogenous, and materials that are more prone to cracking such as dental enamel (Lata, Varghese & Varughese, 2010).

Under neutral and alkaline conditions, CPP molecules increase the calcium phosphate solubility by stabilizing amorphous calcium phosphate (Rose, 2000). 900 ppm fluoride was introduced in the CPP-ACP formulation, favouring remineralization. CPP-ACPF is responsible for the increase in calcium and phosphate concentration, with combined effect of fluoride it incorporates ions in the dental biofilm where fluorapatite (FA) containing stable, remineralising precipitates has been formed (Soares et al., 2017).

According to the manufacturer’s instructions, for maximum benefits the GC Tooth Mousse Plus can be left on the tooth surface as long as possible. So that in this study the excess paste was wiped off after 5 min without rinsing to leave the residues and immersed in artificial saliva.

Bioenamel Remineralising gel is an agent with a main composition of glycerine, potassium nitrate, bioglass, and carbopol effectively enhances the re-mineralization of the enamel and also reduces post whitening sensitivity. Bioglass formulation initially form an octa calcium phosphate (OCP) phase which is found to be a precursor for hydroxyapatite formation. OCP has an attractive remineralization potential that it can incorporate a fluoride source and promotes the transformation of OCP to apatite and also acid-durable fluoridated apatite could be formed (Hill & Gillam, 2015).

When in contact with saliva, bioglass particles react and precipitation is one of the important process that occur to occlude dentine tubules. A calcium and phosphate layer has been formed by the ions released from the glass particles which occludes the dentinal tubules and restrict the dentinal fluid flow. This particular layer is further crystallized to form hydroxyapatite and the hydroxyapatite maturation is brought about by presence of silica (Hench, 2006).

Mitchell, Musanje & Ferracane (2011), and Tirapelli et al. (2011) proposed that so far bioglass particles have been incorporated into toothpastes or they can be applied onto tooth surfaces with any aqueous medium. They adhere to dentin and form a hydroxy carbonapatite layer rapidly, and seals the tubule. Recent studies have stated that comparing with other conventional treatment procedures bioglass seems to be effective in function.

As per manufacturers instructions bioenamel gel could be used in areas of bleaching, Remineralization of tooth structure, in areas of sensitivity, non-carious lesions and after oral prophylaxis. In this present study significant results were obtained in comparison with baseline, after demineralisation and after remineralisation which states that hardness of the enamel surface has been increased.

Jayarajan et al. (2011) stated that fluoride ions when adsorbed onto enamel surface increases remineralisation and prevents dissolution. CPP-ACPF, fluoride concentration (0.2% or 900 ppm of NaF) promotes precipitation of free calcium phosphate and fluoride ions, maintains a level of supersaturation and suppress demineralization. Thus CPP-ACPF is considered to be an acceptable local slow-delivery system to treat the demineralised lesion.

Dhanya et al. (2021) stated that synergistic anticariogenic effects of CPP-ACP and fluoride together showed CPP-ACPF has a greater remineralization action. Elsayad, Sakr & Badr (2009) concluded in their study that combining fluoride with CPP-ACP can give a synergistic effect on remineralization of enamel surface.

This study also shows that on comparing the mean value difference from demineralization to remineralization between group 2 (CPP-ACPF; 8.34) and group 3 (Bioenamel gel; 5.87), the former showed a higher level of remineralization than other.

## Conclusion

When compared to the control group, the two remineralizing agents CPP—ACPF and Bioenamel remineralising gel which were used in this study showed remineralization potential on enamel surfaces by improving the surface microhardness *in vitro*. Although GC Tooth Mousse Plus has shown slightly superior SMH values, when both treatment modalities were compared with each other, there was no statistical significance among them.

## Supplemental Information

10.7717/peerj.14098/supp-1 Supplemental Information 1 Micro hardness number of each sample.

## Additional Information and Declarations

### Data Availability

The following information was supplied regarding data availability: The raw measurements are available in the Supplemental File.
